# New determination of the gravitational constant *G*

**DOI:** 10.1093/nsr/nwz210

**Published:** 2020-02-03

**Authors:** Zheng-Tian Lu

**Affiliations:** Hefei National Laboratory for Physical Sciences at the Microscale, CAS Center for Excellence in Quantum Information and Quantum Physics, University of Science and Technology of China, China

A new and so far the most precise determination of the gravitational constant *G* has been made by Jun Luo and colleagues at the Huazhong University of Science and Technology (HUST) [1].

The combination of *G* with two other most fundamental physical constants, the speed of light *c* and Planck's constant *ħ*, can together construct the natural units (a.k.a. Planck units) of time, length and mass. These relationships inform us that, among the aforementioned three constants and three units, a total of six physical quantities, three and only three quantities should have their values defined. In the SI system, just updated in 2019, the following were chosen to have defined values: the second as the unit of time, *c* and *ħ*. Today, *G* remains to be measured.

*G* lags far behind *c* and *ħ* in measurement precision due to the extreme weakness of gravity. Within a hydrogen atom, the gravitational force between the proton and the electron is weaker than the Coulomb force by 39 orders of magnitude. Measuring *G* in the quantum world is exceedingly difficult due to interference of any residue electromagnetic interaction. So far, the most precise measurements of *G* were made on macroscopic objects using the torsion balance method pioneered by John Michell and Henry Cavendish in the late eighteenth century. For sure, the implementation of the method has been vastly improved with modern ingenuity.

But all was not well in recent years. A sense of confusion hung over the field as over 10 independent measurements of *G* made in the past two decades exhibited discrepancies, in some cases as large as 10 times the quoted uncertainties. The situation cast doubt on the physicist’s ability to measure weak forces on the laboratory scale. Two international meetings were organized in 2014 to examine possible biases in these measurements and to encourage new measurements that might resolve the impasse [2]. The paper of Li *et al.* [1] was just what the community had been waiting for.

**Figure 1.**
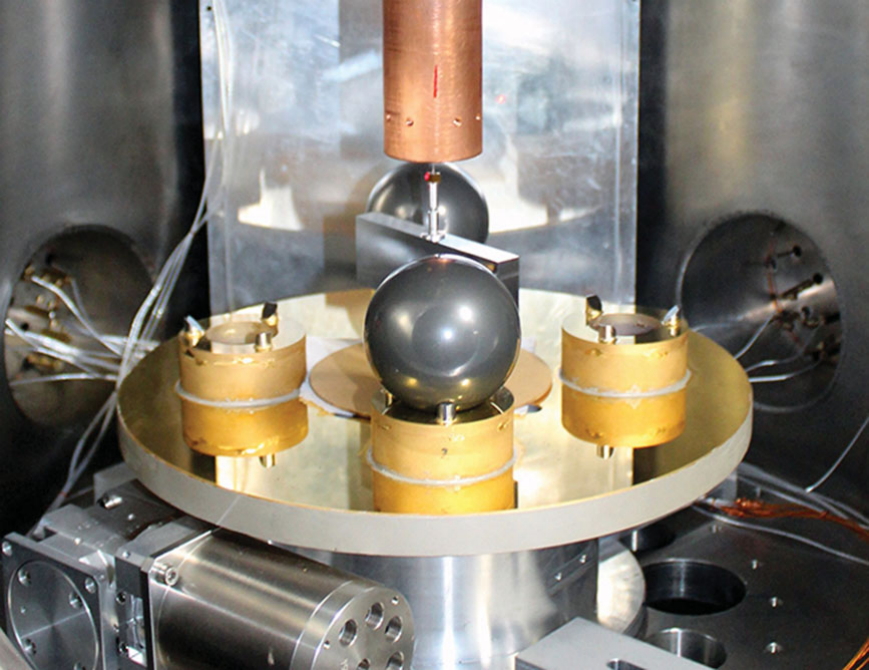
A torsion balance at HUST. Each stainless steel ball has a diameter of 57 mm and a mass of 778 g. Photo credit: Shanqing Yang.

Building upon the experience of a continual quest at HUST for over three decades, Jun Luo and colleagues conducted two types of torsion balance experiments. In one experiment (Fig. 1), the center plate was hung on a thin fiber twisting back and forth. Its oscillation period (∼7 min) was precisely measured to reveal the gravitational pull from the two stationary balls. In another experiment, the balance and the balls revolved around a common center axis, with their rotation speeds varied in a controlled way so that the fiber was maintained in its natural position without any twist. Ideally, these two methods, possessing two different sets of systematic effects, should converge into a consistent value of *G*.

A brilliant example of precision measurement, this work combined both insight and patience in many details. The balance was so close to lossless that a free oscillation could go on for several months on its own, yet its minute deviation from an ideal spring needed to be examined and understood. Each stainless steel ball, with a diameter of 57 mm, was hand polished to guarantee its exact spherical shape allowing deviation of at most 0.25 μm. The measurements were conducted inside a cave laboratory 100 m away from any human presence.

Both methods in this work reached a relative accuracy of 12 ppm, 1000 times more accurate than the original Cavendish value. However, the two methods differ from each other by 45 ppm, suggesting biases yet to be uncovered.

While determining *G* is of fundamental interest in physics, the capability in small force measurements leads to useful technologies. Gravimetry has a wide range of applications including navigation, oil and gas exploration, and water resource management.
